# Extracapsular wide resection of a femoral neck osteosarcoma and its reconstruction using a pasteurized autograft-prosthesis composite: A case report

**DOI:** 10.3892/ol.2013.1503

**Published:** 2013-07-31

**Authors:** TAKETOSHI YASUDA, TAKESHI HORI, KAYO SUZUKI, JUN HACHINODA, ISAO MATSUSHITA, YOSHIAKI ITO, MASAHIKO KANAMORI, TOMOATSU KIMURA

**Affiliations:** 1 Department of Orthopaedic Surgery, Faculty of Medicine, University of Toyama, Toyama 930-0194, Japan; 2 Department of Orthopaedic Surgery, Takaoka City Hospital, Toyama 933-8550, Japan; 3 Department of Orthopaedic Surgery, Toyama Rosai Hospital, Toyama 937-0042, Japan

**Keywords:** extracapsular resection, total hip arthroplasty, osteosarcoma

## Abstract

The requirement for an extracapsular resection is indicated for malignant bone tumors that have disseminated intracapsularly. Extracapsular resections are often performed for malignant tumors arising from the knee joint, but there are relatively few studies that have described an extracapsular resection of a tumor arising from the hip joint. The present study describes a case of extracapsular wide resection of the hip joint using rotational acetabular osteotomy. The patient was a 17-year-old female and the diagnosis was an osteoblastic osteosarcoma with a pathological fracture of the femoral neck. The joint was reconstructed using an allograft-implant composite graft and total hip arthroplasty. Although the patient presented a slight Trendelenburg gait, no recurrence or metastases were identified during a follow-up period of 3 years. The clinical features and surgical procedure of the case are described.

## Introduction

Osteosarcoma (OS) is the most common malignant bone tumor in adolescents and young adults, and is characterized by the proliferation of tumor cells producing osteoid or an immature bone matrix. Despite advances in multimodality treatments, consisting of aggressive adjuvant chemotherapy and wide local excision, pulmonary metastasis occurs in 60–80% of patients with OS and remains a major cause of fatal outcomes (1–3). OS shows a profound propensity for the involvement of the long bones of the appendicular skeleton, in particular, the distal femur and the proximal tibia and humerus. OS also tends to involve lesions of the metaphysis (4,5). The proximal femur is involved in ~5% of all cases of OS (5).

Pathological fractures are present in 5–10% of patients with OS (6). Pathological fractures pose particular problems, as a fracture hematoma, which nearly always contains tumor cells, may either be intracapsular, thus contaminating the joint, or extracapsular, and is usually associated with widespread contamination of the surrounding tissues. When contamination is localized in the intracapsular space, an extracapsular wide resection is an adequate treatment (7). Extracapsular wide resections around the knee joint are common. However, extracapsular wide resections of the hip joint are uncommon due to the rarity of cases of OS in this location. The present study describes the case of a female who was diagnosed with OS of the proximal femur, with a pathological fracture. The patient underwent an extracapsular resection of the hip joint with subsequent reconstruction using a pasteurized autograft-prosthesis composite. The present study describes the clinical course of the patient and the novel surgical procedure that was used to manage the OS with a pathological fracture of the proximal femur. The study was conducted following a clinical research review by the ethics committee of Toyama University Hospital (Toyama, Japan). Informed consent was obtained from the patient, who was advised that the data from the case would be submitted for publication.

## Case report

### Patient

**Figure 1:**
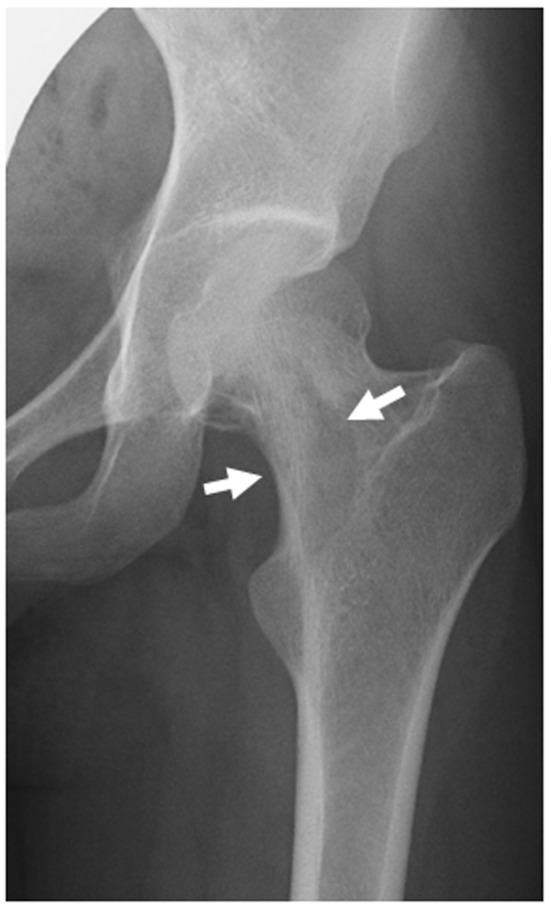
Radiographic observations at the time of the initial consultation showing an osteolytic lesion with an osteosclerotic change to the femoral neck (arrows).

**Figure 2:**
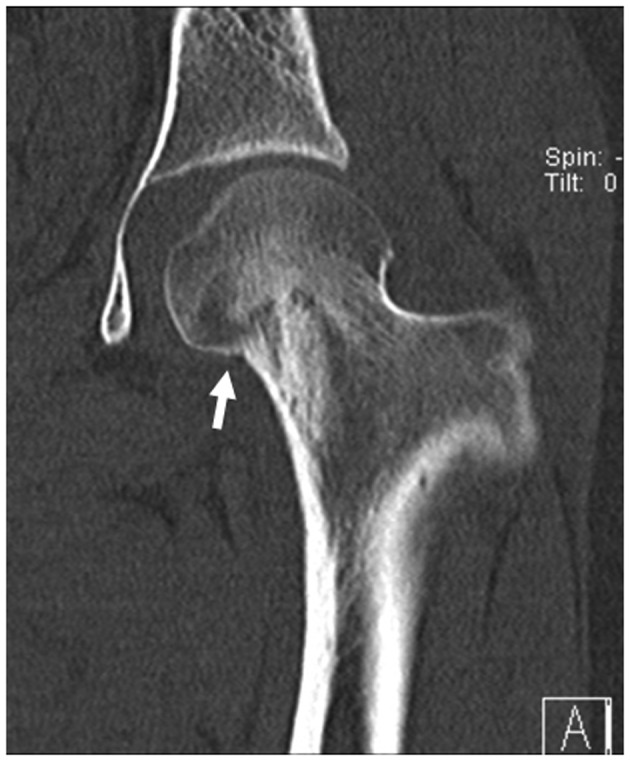
Computed tomography (CT) observations at the time of the initial consultation showing discontinuity of the cortical bone at the medial side of femoral neck (arrow). A periosteal reaction is not shown..

**Figure 3:**
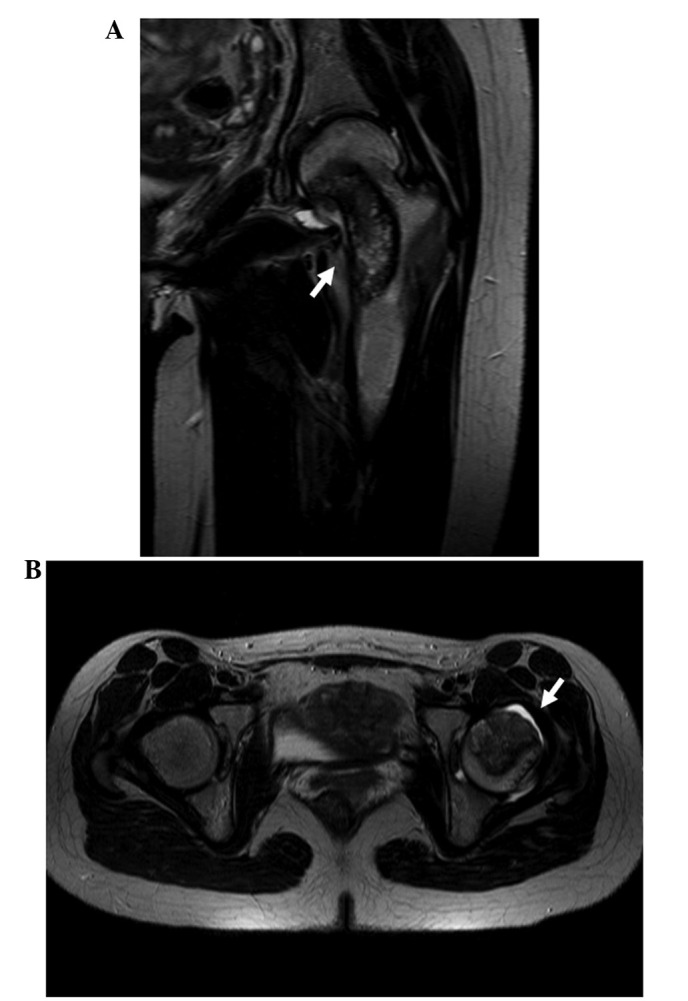
Magnetic resonance imaging (MRI) observations. (A) T2-weighted coronal imaging and (B) T2-weighted axial imaging. MRI showing a heterogeneous tumor arising from the femoral neck and extending to the trochanteric site, which is hypointense compared with the bone (arrow). In the capsule of the hip joint, a high intensity area is apparent (arrow).

**Figure 4:**
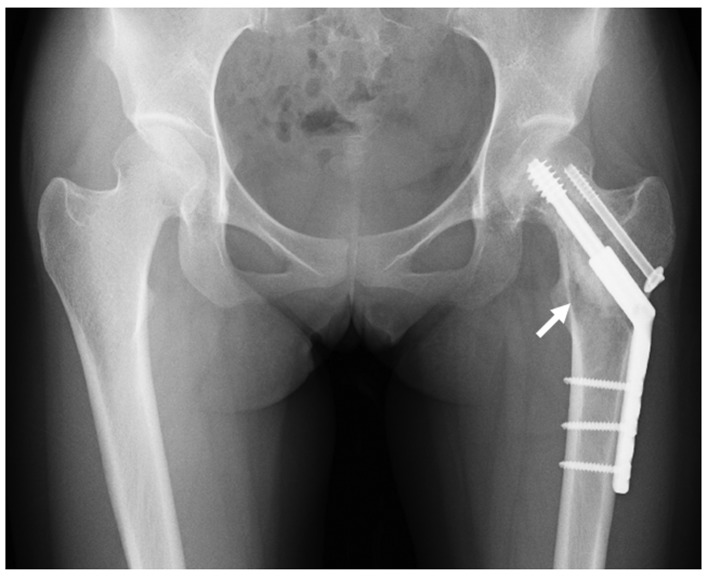
Radiographic observations following the first surgery. The tumor has been curettaged and hydroxyapatite (HA) granules have been transplanted (arrows).

**Figure 5:**
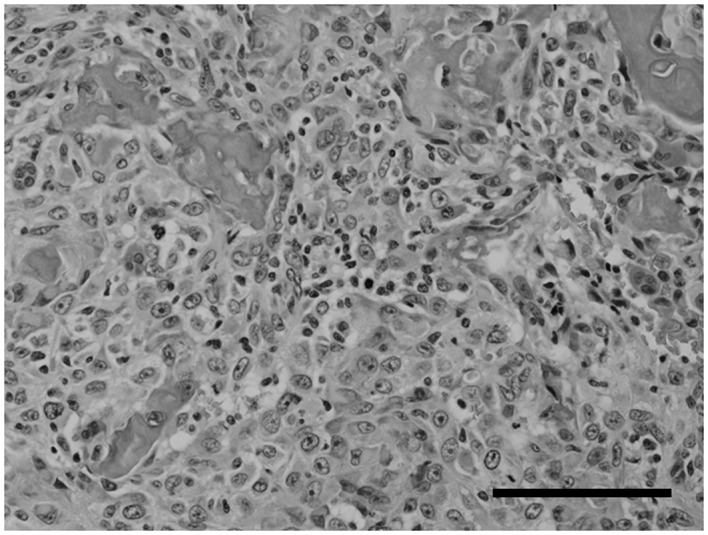
Histological appearance of the open biopsy specimen. Spindle-shaped tumor cells with atypical nuclei have proliferated with the formation of osteoid or immature bone matrix (hematoxylin and eosin staining; scale bar, 100 μm).

A 17-year-old female presented with a three-month history of left hip pain. Plain radiography revealed an osteolytic lesion with an osteosclerotic change to the femoral neck (Fig. 1). A periosteal reaction was not observed. Computed tomography (CT) revealed an osteolytic lesion with an osteosclerotic change and a pathological fracture of the femoral neck (Fig. 2). T2-weighted coronal magnetic resonance imaging (MRI) showed a heterogeneous bone tumor arising from the femoral neck and extending to the trochanteric site, which was hypointense compared with the bone (Fig. 3A). Hyperintensity was observed in the capsule of the hip joint due to hemorrhage (Fig. 3B). Chest radiography and CT revealed no evidence of lung metastasis. The initial differential diagnosis was of a benign bone tumor, including a solitary bone tumor, an aneurismal bone cyst or fibrous dysplasia. An open biopsy was performed. The results from the examination of the intraoperative frozen section were consistent with a fibrous benign tumor. Due to the presence of the pathological fracture, tumor curettage, hydroxyapatite (HA) granule (Apaceram^®^; Hoya Co., Tokyo, Japan) transplantation and internal fixation using a compression hip screw (CHS) system (Omega Plus T1; Stryker Japan Co., Tokyo, Japan) were performed to prevent displacement and avascular necrosis of the femoral neck (Fig. 4). However, a histopathological study of the resected specimen revealed that the tissue contained atypical tumor cells and exhibited a formation of osteoid or immature bone matrix (Fig. 5). Immunohistochemically, the majority of the tumor cells were strongly positive for vimentin and alkaline phosphatase (ALP). Based on these data, a histopathological diagnosis of conventional osteoblastic OS was made.

The patient was administered multiagent chemotherapy, including high-dose methotrexate (HD-MTX; 10 g/m^2^ day 1), cisplatin (CDDP; 120 mg/m^2^ day 1) and adriamycin (ADM; 30 mg/m^2^ day 2), according to the neoadjuvant chemotherapy for osteosarcoma (NECO) 95-J protocol (8). When the neoadjuvant chemotherapy was completed, the response to chemotherapy was evaluated using MRI, CT and plain radiography and a partial response was observed.

**Figure 6:**
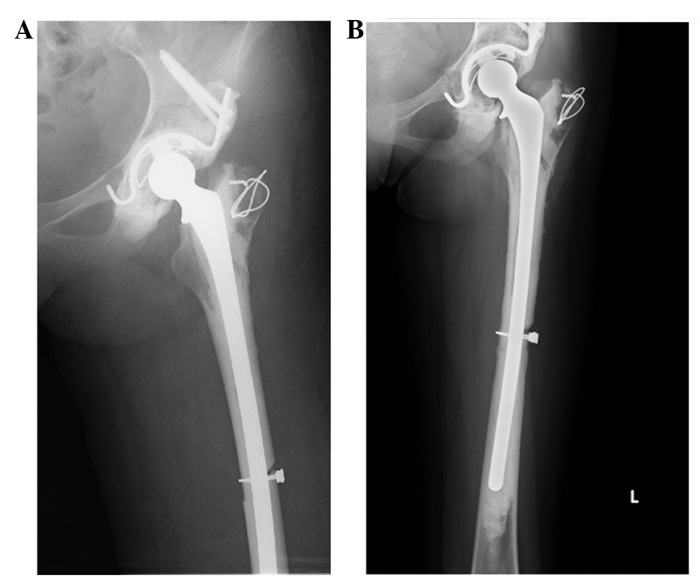
Radiographic observations following the second surgery. (A) Acetabular side and (B) femoral side. The acetabular side has been reconstructed using the pasteurized bone, a Kerboull-type (KT) plate and an acetabular cup with bone cement. The femoral side has been reconstructed using the long stem with bone cement.

The patient subsequently underwent a wide resection and reconstruction using a pasteurized autograft-prosthesis composite, as described later in this study (Fig. 6). The histopathological response to chemotherapy was grade IV according to the criteria of Rosen *et al*(9). The patient was administered adjuvant chemotherapy, as above. No recurrence or metastases were identified during a follow-up period of three years. Radiography revealed a partial union of the medial junction of the distal femur after 1 year (Fig. 6). The functional score, according to the study by Enneking *et al*(10), was 92%. The patient showed a slight Trendelenburg gait due to disorder of the gluteus medius muscle.

### Surgical technique

**Figure 7:**
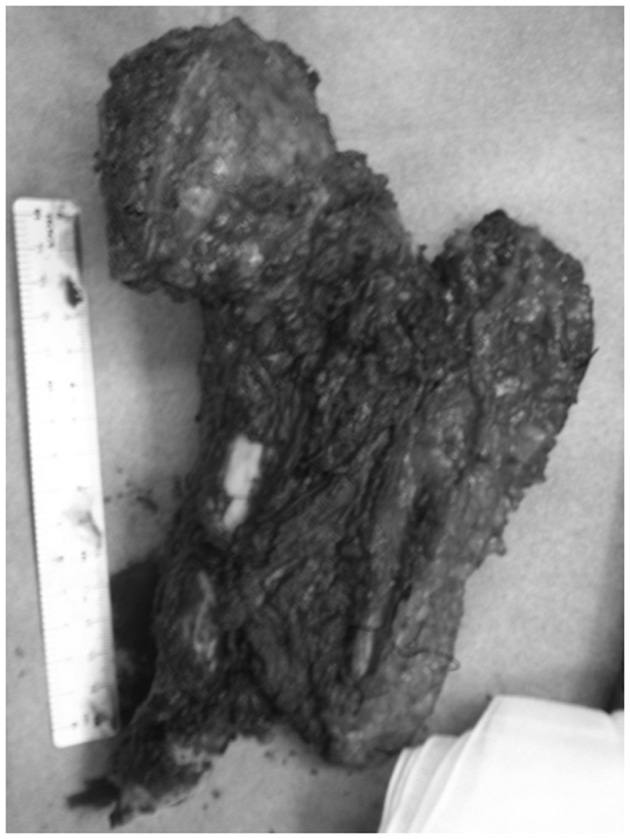
Gross appearance of the surgical specimen. The acetabulum and proximal femur have been resected en bloc without breaking the joint capsule.

The patient was placed in a lateral position. A long posterolateral incision was made to contain the biopsy scar and all the potentially contaminated areas that were included in the scars from the drains. Contaminated areas, including the subcutaneous tissue, iliotibial band and vastus lateralis muscle, were resected and an adequate wide resection of the bone was performed. The distal femur was resected 5 cm away from the tip of the CHS plate. The pelvis was resected according to the rotational acetabular osteotomy (RAO) technique described previously (11). Briefly, the osteotomy was performed using a specially curved osteotome that was designed to approximately correspond to the circumferential curvature of the acetabulum. The line of the osteotomy followed the proximal side of attachment of the capsule to the pelvis. The osteotomy was carried out anteriorly and posteriorly around the circumference of the acetabulum, with the osteotome being allowed to follow its own curve. En bloc resection of the hip joint was performed without breaking the capsule (Fig. 7). Pasteurization of the resected tissue was then performed (12). Briefly, the bone was heated in physiological saline at 65°C for 30 min following curettage of the tumor tissue and intramedullary reaming. The capsule of the hip joint was removed from the bone. The acetabular cartilage was removed from the pasteurized bone using curets and careful reaming, with an attempt to leave the subchondral bone of the acetabulum intact. The pasteurized acetabulum was fixed to the ilium, ischium and pubic bone using a SuperFIXSORB screw (Takiron Co., Osaka, Japan). For further reinforcement of the acetabulum, a Kerboull-type (KT) plate (Japan Medical Materials Corp., Osaka, Japan) was installed. Following the fixation of the graft to the host bone, the pasteurized acetabulum was resurfaced with a polyethylene cup that was cemented in place. The distal side of the pasteurized femur was fixed with wire. Conventional cemented total hip arthroplasty (THA; Stryker Japan, Co.) was performed using an Omnifit Cemented Long Stem. To prevent infection, the bone cement was mixed with antibiotics. Every 40 g of cement contained 1 g vancomycin hydrochloride and 400 mg amikacin sulfate.

## Discussion

In OS, the presence of a pathological fracture at the time of diagnosis (5–10% of all cases) is associated with a poor outcome (13). This may be due to fact that the fracture causes a local hematoma, which facilitates the dissemination of the tumor. Therefore, the presence of a pathological fracture has been considered a contraindication for limb salvage and an indication for an immediate amputation (14). However, studies have suggested that limb salvage surgery for a pathological fracture, if combined with adjuvant chemotherapy, does not increase the risk of local recurrence (15) and does not decrease overall survival compared with amputation (16). In the present study, a pathological femoral neck fracture was recognized and dissemination was limited to the space within the capsule.

In the present study, a periosteal reaction, which is a characteristic finding in OS, was not recognized at the time of the initial consultation, as the main lesion was located in the intra-articular area and not in the periosteum. Furthermore, the imaging findings were consistent with a differential diagnosis of a benign bone tumor, including a solitary bone cyst and fibrous dysplasia. The examination of the intraoperative frozen section showed features of a fibrous benign tumor, probably due to crush artifacts. Therefore, tumor curettage, HA transplantation and osteosynthesis by CHS were performed during the first surgery. This scenario illustrates the limitations of the initial diagnostic measures. However, even when a diagnosis of OS is made at the first surgery, osteosynthesis using a screw may be required to prevent displacement.

In the present case, a resection of the extra-articular hip joint, femoral neck and pubic rami, i.e., a type II resection according to the principles outlined by Enneking *et al*(17) and Bickels and Malawer (18), was adequate. Although several methods for reconstruction following resection have been reported, including pelvic prosthesis arthroplasty (19–21), allograft reconstruction (with or without a total hip prosthesis) (22–25), arthrodesis and pseudarthrosis, a ‘gold standard’ has yet to be established due to poor post-operative function and high complication rates. Regardless of the methods that are used, infection and dislocation are common post-operative complications. Infection rates range from 18–33% in saddle prosthesis arthroplasty (19–21) and 8–60% in allograft reconstruction (with or without a total hip prosthesis) (22–25).

Pasteurization is a method of autograft recycling in which extracorporeal heating of the tumor-bearing bone segment is followed by its reimplantation (26,27). This strategy is a well-established method of reconstruction in certain countries, particularly in Asia and Africa. It is a simple, easily accessible and economical alternative to the usual reconstructive modalities, with comparable and acceptable functional outcomes and complication rates, in addition to its social and religious acceptance in these countries (26,27). However, a variety of complications associated with pasteurized autografts have been described, including graft fracture, collapse, infection, delayed or non-union of the junction and mechanical implant failure (28,29). Although the present patient had a favorable clinical course, longer term follow-up is necessary.

## References

[1] Ferrari S, Palmerini E (2007). Adjuvant and neoadjuvant combination chemotherapy for osteogenic sarcoma. Curr Opin Oncol.

[2] Wittig JC, Bickels J, Priebat D, Jelinek J, Kellar-Graney K, Shmookler B, Malawer MM (2002). Osteosarcoma: a multidisciplinary approach to diagnosis and treatment. Am Fam Physician.

[3] Bacci G, Lari S (2001). Current treatment of high grade osteosarcoma of the extremity: review. J Chemother.

[4] Unni KK, Inwards CY, Bridge JA, Kindblom LG, Wold LE, Silverberg SG, Sobin LH (2005). Chondromyxoid fibroma. AFIP Atlas of Tumor Pathology, Tumor of the Bones and Joints.

[5] Raymond AK, Ayala AG, Knuutila S, Fletcher CDM, Unni KK, Mertens F (2002). Conventional osteosarcoma. World Health Organization classification of tumours, pathology and genetics of tumours of soft tissue and bone.

[6] O'Hara JM, Hutter RV, Foote FW, Miller T, Woodard HQ (1968). An analysis of thirty patients surviving longer than ten years after treatment for osteogenic sarcoma. J Bone Joint Surg Am.

[7] Gebert C, Wessling M, Hoffmann C (2011). Hip transposition as a limb salvage procedure following the resection of periacetabular tumors. J Surg Oncol.

[8] Iwamoto Y, Tanaka K, Isu K (2009). Multiinstitutional phase II study of neoadjuvant chemotherapy for osteosarcoma (NECO study) in Japan: NECO-93J and NECO-95J. J Orthop Sci.

[9] Rosen G, Caparros B, Huvos AG (1982). Preoperative chemotherapy for osteogenic sarcoma: selection of postoperative adjuvant chemotherapy based on the response of the primary tumor to preoperative chemotherapy. Cancer.

[10] Enneking WF, Dunham W, Gebhardt MC, Malawar M, Pritchard DJ (1993). A system for the functional evaluation of reconstructive procedures after surgical treatment of tumors of the musculoskeletal system. Clin Orthop Relat Res.

[11] Ninomiya S, Tagawa H (1984). Rotational acetabular osteotomy for the dysplastic hip. J Bone Joint Surg Am.

[12] Eid AS, Jeon DG, Cho WH (2010). Can bone scintigraphy predict the final outcome of pasteurized autografts?. Skeletal Radiol.

[13] Ferguson PC, McLaughlin CE, Griffin AM, Bell RS, Deheshi BM, Wunder JS (2010). Clinical and functional outcomes of patients with a pathologic fracture in high-grade osteosarcoma. J Surg Oncol.

[14] Jaffe N, Spears R, Eftekhari F (1987). Pathologic fracture in osteosarcoma. Impact of chemotherapy on primary tumor and survival. Cancer.

[15] Natarajan MV, Govardhan RH, Williams S, Raja Gopal TS (2000). Limb salvage surgery for pathological fractures in osteosarcoma. Int Orthop.

[16] Bacci G, Ferrari S, Longhi A (2003). Nonmetastatic osteosarcoma of the extremity with pathologic fracture at presentation: local and systemic control by amputation or limb salvage after preoperative chemotherapy. Acta Orthop Scand.

[17] Enneking WF, Spanier SS, Malawer MM (1981). The effect of the Anatomic setting on the results of surgical procedures for soft parts sarcoma of the thigh. Cancer.

[18] Bickels J, Malawer MM, Malawer MM, Sugarbacker PH (2001). Overview of pelvic resections: surgical considerations and classification. Musculoskeletal Cancer Surgery.

[19] Renard AJ, Veth RP, Schreuder HW (2000). The saddle prosthesis in pelvic primary and secondary musculoskeletal tumors: functional results at several postoperative intervals. Arch Orthop Trauma Surg.

[20] Cottias P, Jeanrot C, Vinh TS, Tomeno B, Anract P (2001). Complications and functional evaluation of 17 saddle prostheses for resection of periacetabular tumors. J Surg Oncol.

[21] Natarajan MV, Bose JC, Mazhavan V, Rajagopal TS, Selvam K (2001). The Saddle prosthesis in periacetabular tumours. Int Orthop.

[22] Bell RS, Davis AM, Wunder JS, Buconjic T, McGoveran B, Gross AE (1997). Allograft reconstruction of the acetabulum after resection of stage-IIB sarcoma. Intermediate-term results. J Bone Joint Surg Am.

[23] Langlais F, Lambotte JC, Thomazeau H (2001). Long-term results of hemipelvis reconstruction with allografts. Clin Orthop Relat Res.

[24] Ozaki T, Hillmann A, Bettin D, Wuisman P, Winkelmann W (1996). High complication rates with pelvic allografts. Experience of 22 sarcoma resections. Acta Orthop Scand.

[25] Yoshida Y, Osaka S, Mankin HJ (2000). Hemipelvic allograft reconstruction after periacetabular bone tumor resection. J Orthop Sci.

[26] Manabe J, Ahmed AR, Kawaguchi N, Matsumoto S, Kuroda H (2004). Pasteurized autologous bone graft in surgery for bone and soft tissue sarcoma. Clin Orthop Relat Res.

[27] Sakayama K, Kidani T, Fujibuchi T, Kamogawa J, Yamamoto H, Shibata T (2004). Reconstruction surgery for patients with musculoskeletal tumor, using a pasteurized autogenous bone graft. Int J Clin Oncol.

[28] Ahmed AR, Manabe J, Kawaguchi N, Matsumoto S, Matsushita Y (2003). Radiographic analysis of pasteurized autologous bone graft. Skeletal Radiol.

[29] Jeon DG, Kim MS, Cho WH, Song WS, Lee SY (2007). Reconstruction with pasteurized autograft-total hip prosthesis composite for periacetabular tumors. J Surg Oncol.

